# Applications of Cell-Penetrating Peptides for Tumor Targeting and Future Cancer Therapies

**DOI:** 10.3390/ph5090991

**Published:** 2012-09-12

**Authors:** Jakob Regberg, Artita Srimanee, Ülo Langel

**Affiliations:** 1 Department of Neurochemistry, The Arrhenius Laboratories for Natural Sciences, Stockholm University, Svante Arrhenius väg 21A, SE-106 91 Stockholm, Sweden; 2 Department of Pharmaceutical Chemistry, Faculty of Pharmacy, Mahidol University, 447 Sri-Ayudhya Rd. Bangkok 10400, Thailand

**Keywords:** cell penetrating peptides, cancer, drug delivery, oligonucleotides, siRNA therapeutics, cell targeting peptides

## Abstract

Cell-penetrating peptides provide a highly promising strategy for intracellular drug delivery. One relevant clinical application of cell-penetrating peptides is cancer therapeutics. Peptide based delivery could increase the uptake of drugs in tumor cells and thereby increase the efficacy of the treatment, either of conventional small molecular drugs or oligonucleotide based therapeutics. This review is focused on the cancer applications of cell penetrating peptides as delivery systems; different aspects of drug loading, cargoes and delivery are discussed together with methods for targeted delivery, activatable cell-penetrating peptides and transducible agents coupled to cell-penetrating peptides.

## 1. Introduction

Cell-penetrating peptides (CPPs) provide a promising solution to the problems commonly related with drug delivery of conventional cancer chemotherapeutics as well as oligonucleotide based treatments. Cell-penetrating peptides are short peptides capable of translocation through the cellular plasma membrane on their own or together with cargoes. CPPs are commonly 5–30 amino acids long, often contain basic amino acid side chains and are in many cases amphipathic. The first CPPs were derived from naturally occurring proteins such as TAT from HIV-TAT [1] and penetratin from the Antennapedia homeodomain [2]. Following this, large numbers of new CPPs, protein-derived as well as designed, have been produced. Increases in efficacy have been observed for chimeric peptides based on known CPPs such as transportan, based on a combination of a galanin and a mastoparan sequence [3], truncated versions known peptides such as transportan 10 (TP10) [4] and chemically modified CPPs using a range of different modifications. The addition of fatty acids, especially stearic acid, has been widely used to increase the cell penetration of CPPs [5]. Synthetic or designed CPPs can range from simple polyarginine structures to more complex peptide sequences designed for helix formation or interaction with cargoes and membranes (see Table 1 for examples of structures).

This ability to transport cargoes over the cellular plasma membrane makes CPPs a promising class of drug delivery vehicles and a large number of different drug-CPP constructs have been synthesized, the cargoes that have been delivered range from classical molecular drugs to different types of oligonucleotides and proteins. The cargoes can either be coupled to peptides by covalent bonds or non-covalent complex formation. Initially, covalent coupling was the most common method, it is still widely used for delivery of small molecular drugs and the staining of CPPs using fluorescent dyes. In the case of large, charged cargoes such as oligonucleotides, non-covalent complex formation is becoming increasingly popular. Using this technique, peptides and cargoes self-assemble into nano-sized complexes by charge interactions and hydrophobic-hydrophilic interactions [6]. Peptide complexes have been used for oligonucleotide delivery in a large number of different systems *in vitro* as well as *in vivo*, the complexation with peptides has been found to increase the stability and serum half-life of oligonucleotide cargoes [7]. Advantages of this method are the relative ease of preparation, that one peptide sequence can be used for a range of different cargoes without chemical modification and that the peptides could potentially shield the cargo from exposure to serum proteins and thereby extend the blood circulation time *in vivo* [8].

**Table 1:** Examples of CPPs.

Peptide	Sequence	Origin	Reference
TAT (48–60)	GRKKRRQRRRQC	Protein-derived	[1]
Penetratin	RQIKIWFQNRRMKWKK-NH_2_	Protein-derived	[2]
pVEC	LLIILRRRIRKQAHAHSK-NH_2_	Protein-derived	[9]
MPG8	AFLGWLGAWGTMGWSPKKKRK-cya	Chimeric	[10]
Transportan	GWTLNSAGYLLGKINLKALAALAKKIL-NH_2_	Chimeric	[3]
Transportan10	AGYLLGKINLKALAALAKKIL-NH_2_	Chimeric, modified	[11]
PepFect3	Stearyl-AGYLLGKINLKALAALAKKIL-NH_2_	Chimeric, modified	[6]
PepFect 6	Stearyl-AGYLLGK(εNH^a^)INLKALAALAKKIL-NH_2_	Chimeric, modified	[12]
PepFect 14	Stearyl-AGYLLGKLLOOLAAAALOOLL-NH_2_	Chimeric, modified	[13]
Polyarginine	R_n_ (n = 6–12)	Designed	[14]
Stearyl-polyarginine	Stearyl-R_n_ (n = 6–12)	Designed	[5]
Pep-1	Ac-KETWWETWWTEWSQPKKKRKV-cya	Designed	[15]
Pep-3	KWFETWFTEWPKKRK-cya	Designed	[16]
CADY	Ac-GLWRALWRLLRSLWRLLWRA-cya	Designed	[17]
YTA2	YTAIAWVKAFIRKLRK-NH_2_	Designed	[18]
YTA4	IAWVKAFIRKLRKGPLG-NH_2_	Designed	[19]
SynB1	RGGRLSYSRRRFSTSTGR	Protein-derived	[20,21]
SynB3	RRLSYSRRRF	Protein-derived	[20]
Maurocalcine	GDCLPHLKLCKENKDCCSKKCKRRGTNIEKRCR	Protein-derived	[22,23]
PTD4	YARAAARQARA	Protein-derived	[24]

cya is cysteamide. ^a^ Lysine tree with trifluoromethylquinoline derivative modifications [12].

**Figure 1:**
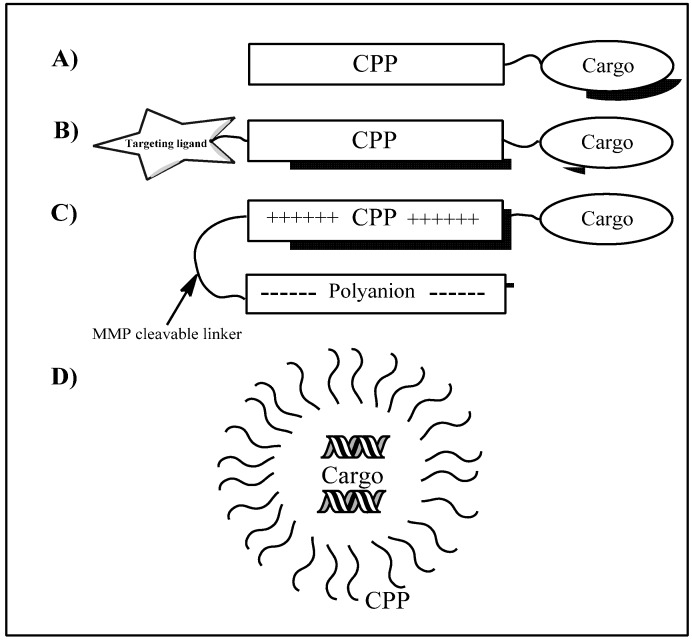
CPP loading and targeting strategies. (**A**) Covalent conjugation of CPP to cargo; (**B**) CPP coupled to targeting ligand and cargo; (**C**) Activatable CPP construct consisting of a peptide, cargo and protecting polyanion with a target specific MMP cleavable linker, after cleavage of the linker the peptide dissociates from the polyanion and becomes an active CPP; (**D**) Non-covalent complex of CPPs and cargo (oligonucleotides or other macromolecules). The complex is formed by electrostatic and hydrophobic interactions between the CPP and the cargo.

The ability to transport cargoes into the cell makes CPP-based delivery a promising strategy for cancer drug delivery. Both classical chemotherapeutics and modern gene-based drugs could potentially be delivered into tumor cells. An additional advantage is the possibility of combining peptide sequences for cell penetration with targeting peptides, thereby creating selective delivery systems. Similarly, activatable CPPs can be obtained by coupling shielding polyanions to the peptide with target-specific cleavable linkers. When this linker is cleaved the peptide becomes an active CPP (see Figure 1 for illustrations of constructs). Cell penetrating peptides could also be used to increase the uptake of other drug delivery systems such as polymer based systems, liposomes, and different types of nanoparticles (not covered in this review).

## 2. Uptake Mechanisms

When CPPs were first discovered they were assumed to penetrate cell membranes by a receptor independent, non-endocytic mechanism. This assumption was to a large extent based on observations of fluorescently labeled CPPs in fixed cells rather than mechanistic studies of the uptake. Since then a number of studies have found evidence of endocytic uptake of different CPPs and many of the initial localization studies were found to be biased by fixation artifacts [25]. Today, most CPPs are considered to be taken up by different endocytic pathways [26,27], in some cases macropinocytosis was found to be the dominating mechanism but clathrin-mediated-endocytosis and caveolin-dependent endocytosis has also been observed. The observation of endocytic uptake led to a number of peptide modifications aimed at increasing the endosomal escape of peptides or peptide-cargo constructs. Fatty acid modifications and hydrophobic amino acid residues could increase peptide-membrane interactions and destabilize the endosomal membrane; another strategy is to add specific endocymolytic groups to the peptide structure. One example of an endocymolytic modification is the fluoroquinone derivatives used in PepFect 6 [12]. Another strategy is to use “proton sponges”, basic molecules that cause an increased influx of protons into the endosome, thereby disrupting the endosomal membrane [28].

Cell surface heparin sulfate proteoglycans have been shown to interact with CPPs at the cell surface and are thought to play an important role in the uptake of several different CPPs [29,30], however, the exact role of proteoglycans in CPP uptake remains unknown. More recently, scavenger receptors were reported to be involved in the endocytic uptake of PepFect CPPs indicating that the uptake is not only endocytosis mediated but in some cases also receptor dependent [31]. Despite the endocytic uptake of many CPPs, there is still evidence for endocytosis-independent, direct membrane penetration of some peptides. Several peptides have displayed uptake at low temperatures which should inhibit energy-dependent endocytosis and the peptide CADY has recently been shown to translocate over plasma membranes via a direct penetration mechanism [17]. In some cases peptides have also been shown to have different uptake mechanisms depending on cargo loading [32].

## 3. The Application of CPPs in Cancer Therapies

During the last decade, the potential of peptides for drug delivery into cells has been highlighted by the discovery of several CPPs [28]. A number of CPP-conjugated therapies (CTTs) show strong promise for clinical efficacy [33] and have been employed to enhance extracellular and intracellular internalization of various small molecules and biomolecules including plasmid DNA, siRNA, oligonucleotide and peptide nucleic acid (PNA) [34]. The lack of cell specificity remains the major drawback for the clinical development of CPPs [35]. Similarly, the major drawbacks with conventional cancer chemotherapy are lack of satisfactory specificity towards tumor cells and poor antitumor activity. In order to improve these characteristics, chemotherapeutic drugs can be conjugated to targeting moieties [29]. There are several strategies to selectively target cancer cells with CPPs conjugated with targeting ligands: Cell targeting peptides, activatable cell-penetrating peptides and transducible agents. Cell targeting peptides are usually obtained by combining a conventional CPP sequence with a tumor targeting peptide, activatable CPPs are designed to be inactive as cell-penetrating peptides until activated by cancer specific proteases (see Figure 1 for illustrations of constructs), and transducible agents are stabilized in hypoxic tumors but are degraded in normal tissues.

### 3.1. Cell-Targeting Peptides

Targeted delivery by cell-targeting peptides (CTPs) with the ability to recognize cancer cells is particularly attractive for cancer therapy [33,36]. The use of these peptides has increased the specificity and efficacy of drug delivery while reducing side effects in a model system [37] (examples of targeting peptides are given in Table 2).

Screening of phage-display libraries has resulted in the discovery of a number of homing peptides that selectively recognize molecular markers on tumor blood vessels [33,36]. One such homing peptide is cyclic peptide PEGA that has previously been shown to accumulate in breast tumor tissue in mice. PEGA peptide conjugated to the cell-penetrating peptide pVEC was taken up by different breast cancer cells *in vitro*. Additionally, the homing capacity of the PEGA-pVEC was conserved *in vivo*, where the conjugate mainly accumulates in blood vessels in breast tumor tissue or breast cancer vasculature, and consequently was taken up by tumor cells [38,39]. Furthermore, the conjugation of the anticancer drug chlorambucil to pVEC-PEGA was shown to increase the drug efficacy over four times [38], thereby reducing clonogenic survival of MCF-7 cells [39]. In addition, FITC-labelled pVEC-PEGA was internalized into MDA-MB-231cells, but to a lesser extent than FITC-pVEC. FITC-pVEC-PEGA was localized to MDA-MB-435 tumor xenografts after intravenous injection in mice, whereas uptake of FITC-pVEC without PEGA was not only observed in the tumor, but also in the lungs, liver, and skin [39].

The linear five amino acid long peptide CREKA, which was identified in breast tumors in MMTV-PyMT transgenic mice, is another example of a homing peptide [33,36]. The CREKA peptide recognizes clotted plasma proteins and selective homes to tumor blood vessels and stroma, where it binds to fibrin-like structures but it has not been shown to internalize into tumor cells [33,36,39]. In one study, CREKA was used in combination with the CPP pVEC, as a chimeric peptide [33,40]. This new peptide, CREKA-pVEC, is more convenient to synthesize and moreover it is more efficient in translocating cargo molecules into cancer cells as compared to previous published PEGA-pVEC peptides [36]. A recent study demonstrated that CREKA-pVEC is a suitable vehicle for targeted intracellular delivery of a DNA alkylating agent, chlorambucil, as the chlorambucil-peptide conjugate was significantly more effective at killing cancer cells *in vitro* than the anticancer drug alone [33].

Successful *in vivo* transvascular delivery of siRNA to the central nervous system (CNS) of mice was reported by using a synthetic chimeric peptide consisting of a rabies virus glycoprotein (RVG) peptide and a polyarginine CPP. RVG is a 29-amino acid peptide that specifically binds to the acetylcholine receptor expressed by neuronal cells. In order to enable siRNA binding, a chimeric peptide based on RVG with nonamer arginine at the carboxy terminus was synthesized [37,41]. Remarkably, it was found that robust protection from lethal infection was achieved after treatment of mice with RVG-9R/siRNA complexes that target the Japanese encephalitis virus. Although no cancer-related application has been reported, the system has the potential to target brain tumors provided that cell-specific targeting within the CNS can be achieved [37].

The CXC chemokine receptor 4 (CXCR4) is known to be over-expressed in 20 different types of cancer, including prostate, breast, colon, and small-cell lung cancer. In order to target tumor cells that overexpress the CXCR4 receptor, a CXCR4 ligand, DV3, was attached to TAT and two transducible anticancer peptides: A p53-activating peptide (DV3-TAT-p53C′) and a cyclin-dependent kinase 2 (Cdk2) antagonist peptide (DV3-TAT-RxL). This resulted in an enhancement of tumor cell killing compared with treatment with nontargeted parenteral peptides. The treatment was more than twice as effective as unguided CPPs in killing CXCR4 expressing Namalwa lymphoma cells [33,42]. In contrast, there was no difference between DV3 targeted peptide and non-targeted, parental peptide treatment of non-CXCR4-expressing tumor cells. These observations showed that a multidomain approach can be used to further refine and enhance the tumor selectivity of biologically active, transducible macromolecules for treating cancer [42].

In another study, the target ligand folic acid (FA) and the cell penetrating peptide octaarginine (R8) were coupled with the gene vector (PEI (600)-CyD, PC) composed of β-cyclodextrin (β-CyD) and low-molecular-weight polyethylenimine (PEI, Mw 600) to form nano-vectors for highly efficient gene delivery to tumor cells [33]. The resultant ternary nano-complexes of FA-PC/R8-PC/pDNA produced excellent gene transfection abilities in folate receptor-positive tumor cells *in vitro* and *in vivo* due to the combination of folic receptor-mediated endocytosis (associated with FA) and transmembrane functionality (associated with R8) together [33,43].

A cell specific approach to intracellular or intranuclear targeting is to construct CPPs with antibody derivatives or targeting peptides. The first study to report the use of a CPP-targeting label construct reported radiolabeled peptides containing the DEVDG sequence which is selective for downstream caspases such as caspase-3. The study was based on radioiodinated TAT_57-49_-yDEVDG, but only showed a mere two-fold higher uptake in apoptotic cells compared to normal controls [39,44]. In addition, the study of radiolabeled TAT-antibody complexes reported that TAT-peptide conjugated anti-p21 antibodies. Radioiodinated TAT-peptides were site-specifically conjugated to the Fc tail of IgG. These radioimmunoconstructs were shown to internalize into breast cancer cells, and translocate to the nucleus (since the TAT-peptide sequence includes a nuclear localization sequence), where it could be bound to p21, a cyclin-dependent kinase inhibitor and regulator of the cell cycle. In cells exposed to Endothelial Growth Factor (EGF), p21 was upregulated, and ^123^I-anti-p21-TAT retention was increased by 50%. *In vivo*, however, the direct radioiodination of the construct resulted in low stability [39].

**Table 2:** Examples of targeting sequences coupled to CPPs for specific delivery to tumors or tissues.

	Targeting peptide	Active sequence	Targets	References
PEGA		CPGPEGAGC	Tumor blood vessels	[38,39]
CREKA		CREKA	Tumor blood vessels and stroma	[33,36,39,40]
RVG		YTIWMPENPRPGTPCDIF-TNSRGKRASNG	Acetylcholine receptor in neuronal cells	[37,41]
DV3		LGASWHRPDKG	CXC chemokine receptor 4 (CXCR4)	[33,42]
DEVDG		DEVDG	Caspase 3	[39]
ACPP-MMP-2/9		PLGLAG	Proteases in human fibrosarcoma	[45]
ACPP-MMP-2		IAGEDGDEFG	Proteases in breast cancer cells	[19]

### 3.2. Activatable CPPs

One solution to the problem of non-specificity of CPPs is activatable CPPs (ACPPs) [39]. ACPPs are novel *in vivo* targeting agents and also a new class of promising molecular imaging probes for the visualization of enzymatic reactions; they comprise of a polycationic cell-penetrating peptide (CPP) connected via a cleavable linker to a neutralizing polyanion [46,47]. This structure reduces the overall charge to nearly zero and inhibits electrostatic interactions and thereby inhibits uptake into cells [33]. Thus, the cell-penetrating function of a polycationic peptide is efficiently blocked by intramolecular electrostatic interactions with a polyanionic peptide. Proteolysis of a cleavable linker between the polycationic cell-penetrating peptide and the polyanionic peptide affords dissociation of both domains and enables the activated cell-penetrating peptide to enter cells [47]. The linker connecting the polyanionic and the polycationic domains is dissociated by the specific proteases, thereby enabling cell-penetration [48]. The specific proteases can be used to target enzymes associated with cancer and could also have broad applicability to other pathologies where extracellular enzymes play important roles [46].

Cancer associated proteases (CAPs) have recently gained attention as a new method of tumor targeting. CAPs are a set of proteases that are usually absent from or present at very low concentrations in healthy tissues but are often highly up-regulated in cancerous tissues. Some of extensively studied CAPs include urokinase plasminogen activator (uPA), several the matrix metalloproteases (MMPs) and some of the cathepsins. MMPs are probably the most studied CAPs for tumor-responsive drug delivery [37], they are a family of proteolytic enzymes [49,50] which play a major role in tumor invasion [50] and metastasis [49]. MMPs are mainly produced by host stromal cells in most carcinomas and their expression implies a close co-operation between tumor and stromal cells [50]. Many MMPs can be expressed by tumor cell themselves and are regarded as major molecules assisting tumor cells during metastasis [50,51]. Thus, MMPs provide a promising target for drug targeting delivery system to tumor cells. Furthermore, theACPP strategy could also be used to modify antitumor agents for tumor-targeting therapy since MMPs are one type of cleavable enzymes that are associated with tumor diseases [45] and have shown over expression in many forms of human tumors [37].

Several studies have demonstrated a relationship between increased MMP expression and poor clinical outcome in a number of cancers including breast (MMP-11), colon (MMP-1), gastric (MMP-2 and MMP-9), non-small cell lung cancer (MMP-13), esophageal (MMP-7), and small-cell lung cancer (MMP-3, MMP-11, and MMP-14). In addition, the expression of specific MMPs has served as both a prognostic indicator of clinical outcome and a marker of tumor progression in a wide range of tumor types [37].

The synthesis of a conjugate of ACPP with the antitumor drug doxorubicin (DOX) sensitive to matrix metalloproteinase-2 and -9 (MMP2/9) has been used for tumor-targeting therapy purposes. The ACCP-DOX conjugate could be triggered by MMP-2/9, which enabled the activated CPP-DOX to enter cells. The ACCP was designed including three units: A cell-penetrating domain (polyarginine, R9), a cleavable enzyme-specific substrate domain of MMP-2/9 (PLGLAG), and an attenuating peptide domain (DGGDGGDGGDG). PLGLAG was considered a remarkably sensitive sequence, cleaved by MMP-2 and MMP-9 [45]. To prevent CPP distribution toward normal cells, polyanional DGGDGGDGGDG was added to the molecule as an attenuating or shielding motif. As a result, the ACPP-DOX delivery system was temporarily inactive in circulation and in tissues non- or under-expressing MMP. The construct was subsequently activated by overexpressed MMP-2/9 in targets where cargo could be released. Moreover, the proteolytic activation of ACPP-DOX occurs in an enzyme concentration-dependent manner. The enhanced cellular uptake and antiproliferative activity of ACCP-DOX was observed after MMP-sensitive activation and revealed that ACCP-DOX has effective targeting ability for tumor cells rich in MMP-2/9, HT-1080 (Human fibrosarcoma). This promising approach may be a potential prodrug delivery system used to carry antitumor drugs for MMP-related tumor therapy [45].

A new peptide, NoPe (for “no cellular penetration”), is a chimeric peptide which is based on a known CPP, YTA4, with the addition of an inactivating domain and a protease specific linker to MMP-2 to achieve the selective delivery. NoPe is an inactive pro-form of YTA4 and it can be selectively cleaved and activated by MMP-2. In the study, peptide conjugates of fluoresceinyl carboxylic acid and a cytostatic agent MTX were activated by recombinant MMP-2 *in vitro* and the fluoresceinyl-NoPe is selectively accumulated in the tumor tissue in MDA-MB-231 tumor bearing mice after intravenous injection. This strategy proved to be successful for *in vivo* imaging [19].

### 3.3. Transducible Agents of CPPs

Although a number of lethal tumors are treated by local administration of chemotherapeutics, many tumors are disseminated throughout the body, thus necessitating the systemic delivery of anticancer agents. Early *in vivo* experiments demonstrated that TAT proteins are delivered to a large number of organs after intraperitoneal (IP) injection, suggesting that systemic delivery to a primary tumor or to multiple metastases should be feasible with transducible agents [52]. In order to develop a potential therapeutic protein drug highly specific for solid tumors, a fusion protein selectively stabilized in hypoxic tumor cells was constructed [53].

The genetic alterations in tumor cells directly cause the deregulated proliferation and the high metabolic demands of tumor cells, which in turn lead to the development of hypoxia in solid tumors [54]. Human solid tumors contain hypoxic regions that have considerably lower oxygen tension than normal tissues. These impart resistance to radiotherapy and anticancer chemotherapy, as well as predisposing an increase in tumor metastases [53,54]. Therefore, tumor hypoxia has been recognized as a tumor specific microenvironment [54]. A transcriptional factor hypoxia inducible factor-1 (HIF-1), a master regulator of the hypoxic response [33], induces various genes related to angiogenesis and glycolysis, and leads to invasive and metastatic properties in tumor cells [54]. HIF-1 is itself regulated through the oxygen-dependent degradation domains (ODD) of its α-chains (HIFα) [33]. A model fusion protein, oxygen-dependent degradation (ODD)-*β*-galactosidase (*β*-Gal), composed of a part of the ODD domain of hypoxia-inducible factor-1α fused to *β*-Gal. When ODD-*β*-Gal was further fused to the HIV-TAT protein transduction domain (TAT_47-57_) and IP injected to a tumor-bearing mouse, the biologically active fusion protein was specifically stabilized in solid tumors but was hardly detected in the normal tissue [53]. Only the hypoxic regions of the tumors showed evidence of TAT-ODD-*β*-Gal protein. By contrast, TAT-*β*-galactosidase protein could be detected throughout tumors after IP delivery [45]. Furthermore, when the wild-type (WT) caspase-3 (Casp3^WT^) or its catalytically inactive mutant was fused to TAT-ODD and IP injected to a tumor-bearing mouse, the size of tumors was reduced by the administration of TAT-ODD-Casp3^WT^ but not by TAT-ODD-mutant Casp3 [53]. This was able to reduce tumor growth without causing the toxic side effects that would be expected from delivering active caspase-3 to an entire mouse. Thus, it is possible to use functional domains to modulate the type of tissue in which TAT-fusion proteins are active and to, in this way, increase their tumor specificity [52].

## 4. Drug Loading

Numerous CPPs have been described so far, they can be grouped into two major classes, the first requiring chemical linkage with the drug for cellular internalization and the second involving formation of stable, non-covalent complexes with drugs. CPPs constitute very promising tools for the non-invasive cellular delivery of cargo and have been successfully applied for *in vitro* and *in vivo* delivery of therapeutic molecules varying from small molecules, nucleic acids, proteins and peptides to liposomes, and nanoparticles [15] (see Table 3 for examples of drug cargoes).

### 4.1. Small Molecules

Several publications have reported that CPP-DOX conjugates displayed excellent therapeutic efficacy for tumor therapy [45]. Since doxorubicin (DOX) is a drug commonly used to treat various types of cancers, DOX conjugated with CPPs (DOX-CPPs) have been evaluated in terms of apoptosis induction [33]. DOX-CPPs were found to cause apoptotic death in MDA-MB-231, a breast cancer cell line [33,45]. Even though, DOX is a widely used antineoplastic agent in the treatment of several cancers, DOX is restricted to enter the brain through blood-brain barrier (BBB), which is formed by the tight endothelial cell junctions of capillaries within the brain. In addition, the ATP-dependent efflux pump P-glycoprotein (P-gp), first described as participating in the multidrug resistance (MDR) mechanisms of tumor- cell drug resistance, has been shown to be present at the luminal site of the endothelial cells of the BBB. As the result, P-gp may restrict the brain entrance of cytotoxic drugs [20,21]. To overcome the limited entry of DOX to the brain, novel, short naturally derived peptides with the ability to cross the BBB were used to be vectors for drug delivery. DOX was coupled covalently to two peptides, D-penetratin and SynB1. Upon coupling DOX to either D-penetratin or SynB1 vectors, the uptake was increased and led to 20-fold increase in the amount of DOX transported into brain parenchyma [21]. Another study showed SynB3, a truncated derivative of SynB1, giving similar enhancement of DOX brain uptake as SynB1. In addition, both D-SynB3 and L-SynB3 increased the brain uptake of DOX with the same efficiency since the mechanism of transport is non-stereospecific and all this data indicated that the mechanism for the transport of DOX-SynB is unlikely to be via receptor-mediated transcytosis [20]. 

In a study of Maurocalcine (MCa), a 33-mer toxin derived from the venom of the Tunisian scorpion (*Scopio maurus palmatus*) which was hypothesized to also act as a CPP, was covalently coupled to DOX and delivered into MCF-7 and MDA-MB-231 cell lines. The cytotoxicity of this complex comparatively to DOX was studied; the results obtained indicate that MCa is a good peptide vector for the cell entry of DOX and that the coupling strategy does not prevent DOX cytotoxicity. In addition, coupling of DOX to CPPs permits the construct to overcome the DOX resistance of MDA-MB-231 cells [22]. Another amphipathic peptide, CADY-1, is able to form complexes by self-assembly. This distinct characteristic of CADY-1 showed a potent cell-penetrating drug delivery system by forming a stable complex with DOX in a self-assembling manner. This formation extended the blood residence time of DOX similarly to a commercial liposomal DOX formulation. Additionally, the complex was capable of carrying DOX across the cell membrane, thereby increasing the therapeutic index of DOX. The experimental animals treated with a CADY-1/DOX complex exhibited better tolerance and anti-tumor activity than animals treated with either liposomal DOX or the free form of DOX [55].

In another study, two cell-penetrating peptides, penetratin and TAT, were chemically conjugated to DOX. The cytotoxicity, intracellular distribution and uptake were accessed in Chinese Hamster Ovarian carcinoma cells (CHO cells), Human Umbilical Vein Endothelial Cells (HUVEC), differentiated NG108.15 neuronal cell and breast cancer cells MCF7 drug-sensitive or MDA-MB-231 drug-resistant cell lines. The conjugates showed different cell killing activity and intracellular distribution pattern in comparison to free DOX. Treatment with DOX-CPPs, increased the DOX cytotoxicity in CHO cells, HUVEC cells, and MDA-MB-231 cells. However, cytotoxicity was decreased in NG108.15 cells and MCF7. Furthermore, the study of the uptake to both DOX and DOX-CPPs by FACS indicates that CHO, HUVEC, and MDA-MB-231 cell lines accumulate significant amounts of DOX-CPPs compared to DOX alone. Both TAT and penetratin markedly increased the uptake of DOX in CHO, HUVEC, and MDA-MB-231 cells and reduced the extrusion of the drug. In conclusion, DOX-penetratin was the most efficient DOX-CPP conjugate leading to an increased uptake as well as increased cytotoxicity [56].

**Table 3:** Examples of CPPs and drug cargoes for tumor therapy.

CPPs		Method	Cargoes	Application	References
TAT, Penetratin, SynB1		Covalent coupling	DOX	Breast cancer cell lines MDA-MB-231	[20,21,22]
CADY		Non-covalent complex	DOX	Increased therapeutic index and blood residence time	[55]
R9PLGLAGDG-GDGGDGGDG		Covalent, activatable	DOX	Targeting ability to tumors rich MMP-2/9	[45]
YTA2	Covalent coupling		MTX	Resistant breast cancer cells MDA-MB-231	[57,58]
YTA4	Covalent coupling		Fluorescein and MTX	Breast cancer cells MDA-MB-231	[19]
TAT	Covalent coupling		p53	Rabbit eyes harboring human retinoblastoma xenograft	[33,52,54]
Antp	Non-covalent complex		p16	Pancreatic cancer	[33,52,57]
TAT, Antp	Non-covalent complex		Smac	Proapoptotic stimuli	[33]
R8	Non-covalent complex		SmacN7	Reversed apoptotic resistance	[54]
Antp, TAT	Covalent coupling		shepherdin	Caspase-dependent apoptosis	[33]
PTD4	Covalent coupling		Peptide D1, D3, and K4	Antiproliferation effect on cancer cell lines	[24]
MPG8, PEP3	Non-covalent complex		siRNA, PNA	Promotes cellular uptake in cancer cell lines	[10,33]
TP10	Covalent coupling		PNA	Promotes cytosolic delivery	[57]
Penetratin, Transportant	Non-covalent		siRNA	Luciferase and GFP transgenes inhibitor	[57]
cholesteryl-R9	Non-covalent		siRNA	VEGF inhibitor	[57]

Methotrexate (MTX), an anticancer agent with limited use due to resistance problems, prevents tumor proliferation by impairing the synthesis of purine nucleotides, the building blocks of DNA through inhibition of the enzyme dihydrofolate reductase in the cytoplasm. A conjugate of MTX to a CPP, YTA2 (MTX-YTA2), was studied in a model system of MTX resistant breast cancer cells; the cell line MDA-MB-231 was obtained from a patient who acquired MTX resistance during chemotherapy. This cell line lacks expression of reduced folate carrier (RFC), which is the major route for the cellular uptake of MTX. Hence, the tumor cells were defective in transporting MTX and less sensitive to MTX toxicity. In a cell viability study, the EC_50_ values of MTX-YTA2 were five times lower than the values for MTX alone. Consequently, MTX can be successfully delivered into tumor cells by YTA2 and the conjugate can overcome the MTX resistance model of breast cancer cell line MDA-MB-231 [58].

The chemical nuclease metalloporphyrin (manganese (III) porphyrin) can cleave DNA irreversibly and thus constitutes a potential antitumor drug. However, these molecules show low permeability to cell surface membranes. The conjugation of an amphipathic carrier peptide to porphyrin was reported to considerably improve its cellular delivery [59]. Metalloporphyrin linked to MAP was shown to follow the nuclear pathway via a mechanism involving genomic DNA cleavage and was 100-fold more efficient than free metalloporphyrin at inducing tumor cell death [57,59]. To boost the nuclear delivery of a DNA intercalator based on a rhodium complex [57], the conjugate of rhodium-D-octaarginine was studied and showed rapid and efficient concentration of the conjugate containing the metal complex tethered to the CPP in the nucleus of HeLa cells. These results established a clear strategy for targeting octahedral metal complexes inside cells. Tethering of an octaarginine as a CPP onto an ancillary ligand of the metal complex offers a reliable means of intracellular delivery while maintaining the targeting of mismatched DNA sites [57]. 

Although a proof-of-concept is established for delivery of small molecules covalently linked to CPPs, attaching CPPs to small molecules may not be the optimal method for the delivery of all types of therapeutic molecules. Covalent coupling to a peptide could interfere with the function of biologically active molecules, such as oligonucleotides and proteins, or cause steric hindrance of drug binding to targets. Thus, for macromolecule cargoes, non-covalent CPP complexes might provide a better drug delivery solution.

### 4.2. Macromolecules

The development of CPP-fused anticancer macromolecules mediated protein therapy have been extensively studied *in vivo*. A TAT peptide derived from the N-terminus of p53 [33,52] has been used in the application of several tumor suppressor and apoptotic genes. The gene encoding the tumor suppressor p53 is the most common anti-apoptotic lesion in cancer cells and approximately 50% of human cancer bear p53 gene mutations [54]. The N-terminal region of the p53 protein was fused to the TAT (TAT-p53N) leading to induction of a rapid accumulation of p53 and the activation of apoptotic genes [54]. Injection of the peptide into rabbit eyes harboring human retinoblastoma xenografts resulted in a high level of tumor cell apoptosis without significant toxicity to surrounding normal tissue [33,52,54]. Two other reports have shown that systemic delivery of cell-penetrating peptides can be used to inhibit specific tumors *in vivo*. In the first case, TAT was fused to a peptide derived from the VHL tumor suppressor gene that inhibits insulin-like growth factor-I receptor (IGF-IR) signaling in renal cell carcinomas (RCC) [33,52,54]. IP administration of TAT-VHL peptide slowed the growth of subcutaneous RCC tumors in nude mice, primarily through inhibition of cell proliferation, rather than by the induction of apoptosis. A second study found that IP delivery of an Antp-p16 fusion peptide moderately inhibited the growth of pancreatic cancer cells growing as peritoneal and/or subcutaneous tumors in nude mice [33,52,57]. Furthermore, the second mitochondria-derived activator of caspases (Smac) was identified as the protein that is released from the mitochondria to the cytosol in response to apoptotic stimuli and antagonizes inhibitors of apoptosis proteins (IAPs) to promote apoptosis [54]. The studies found Smac-TAT or Smac-Antp sensitized cells to pro-apoptotic stimuli [33] and demonstrated SmacN7 peptide fused to the cell membrane permeable polyarginine (SmacN7R8), which strongly reversed the apoptosis resistance and displayed a synergistic effect with chemotherapy *in vivo* [54].

A recent study utilized a CPP conjugated to an anti-p21 antibody in order to sensitize cancer cells to the DNA damaging from effects of radiation and chemotherapy since blocking p21 nuclear translocation could inhibit the ability of p21 to convey chemo-resistance, resulting in sensitizing cancer cells and allowing for a reduction in chemotherapy dosages. TAT-anti-p21 inhibited the translocation of p21 into the nucleus resulting in the loss of p21 dependent anti-apoptotic effect and to sensitize breast cancer cells to both γ-radiation and camptothecin, a topoisomerase inhibitor [33].

A recently approved patent utilized a p65 derived peptide conjugated with Antp to inhibit p65 from activating transcription. The Antp-p65-1 treated leukemia cells also showed an increase in the cytotoxic sensitivity following exposure to a chemotherapeutic agent, DOX and these conjugated peptide can be used to sensitize chemotherapy resistant cancer cells to apoptosis. Furthermore, an interesting peptide, shepherdin, was designed to mimic a small domain of the survivin protein, that interacts with the ATPase domain of Hsp90, and this peptide was conjugated with CPPs Antp or Tat. Shepherdin caused caspase-dependent apoptosis and loss of membrane integrity. In addition, shepherdin treatment of breast and prostate tumors grown superficially in immune-compromised mice led to a decrease in tumor growth compared to controls [33].

A study of novel chimeric peptides, protein transduction domain 4 (PTD4) conjugated to protein complexes, cyclinD/CDK4, key regulators of the cell progression, showed significant anti-proliferation effects on cancer cell lines. These chimeric peptides, PTD4-D1, PTD4-D3, and PTD4-K4, showed the proliferative inhibition of human esophageal carcinoma cells, breast cancer cells, murine hepatoma cells, and sarcoma cells. In addition, these peptides could induce cell cycle arrest at G1/S phase and apoptosis of cancer cells. *In vivo* the peptides displayed tumor targeting and potent antitumor effects [24].

Cell-penetrating peptides constitute very promising tools for the non-invasive cellular import of oligonucleotides and analogs [33]. Gene regulation at the RNA level using siRNA, ribozymes or antisense oligonucleotide analogues does not require nuclear uptake, however cellular uptake is necessary but limited by the negatively charged nature of the oligonucleotides [57]. Recently a non-covalent strategy has been described based on short amphipathic peptides (MPG8/PEP3) that successfully applied *ex vivo* and *in vivo* for the delivery of therapeutic short interfering RNA (siRNA) and peptide-nucleic acid (PNA) molecules. PEP3 and MPG8 form stable nanoparticles with PNA and siRNA respectively, and promote their efficient cellular uptake, independently of the endosomal pathway, into a wide variety of cancer cell lines without any cytotoxicity [33].

The study of the interaction between RNA and RNA binding protein, a PNA linked to transportan 10 promoted the cytosolic delivery of the PNA. siRNA against vascular endothelial growth factor (VEGF), a multifunctional angiogenic growth factor, was complexed with cholesteryl-R9 and was shown to enhance tumor regression efficacy of the siRNA both *in vitro* and *in vivo*. Thiol containing siRNA against luciferase and GFP transgenes, were conjugated to penetratin and transportan by a disulfide linkage and were shown to efficiently reduce transient and stable expression of the reporter genes in several cell types. The disulfide link would be reduced in the cytoplasm to release the bioactive siRNA. It can be concluded that by using non-covalent CPP-oligonucleotide complexes or conjugates of oligonucleotides to CPPs significant intracellular delivery and biological activity can be achieved [57].

## 5. Future Aspects

After several years of research, CPPs finally seem to be ready to make the transition from lab bench to clinical applications. The cancer therapeutics field will most likely be one of the first fields to benefit from the use of CPPs due to the current lack of clinically relevant delivery systems and the severe side effects of many therapies. Initial CPP systems in clinical use will likely be formulations of standard cancer chemotherapeutics similar to the liposomal formulations already in the market. Following this, targeted delivery systems, activatable CPP constructs and treatments using new therapeutic targets could be developed. CPPs are highly promising candidates for therapeutic delivery of siRNA, in theory one single efficient peptide sequence could be used to deliver hundreds of different siRNAs, enabling therapeutic targets as well as the possibility of personalized cancer treatments. Using simple, non-covalent complexes of siRNA and CPP, the drug constructs could be assembled using a selected siRNA or combinations of several different siRNAs for each patient or cancer type.

Given the high costs of bringing a drug to clinical use, the most common cancer types such as breast and lung cancer are the main candidates for new treatments but the advantages of CPP based therapies might also lead to applications in other cancer forms. The low toxicity of peptide based delivery systems will be a key advantage of this treatment option, when compared to many other means of drug delivery such as free drugs or liposomes. CPPs might also play a role in combined delivery systems based on other types of nanoparticles or on liposomal formulations. Nanoparticles modified with CPPs could potentially have increased cellular uptake, enable crossing of the blood-brain barrier or, in the case of combined penetrating/targeting peptides, display increased specificity of delivery. Altogether, the future for CPPs looks bright and the first CPP-drugs will probably reach the market within the next few years.
